# Coarse to Fine Audio-Visual Size Correspondences Develop During Primary School Age

**DOI:** 10.3389/fpsyg.2019.02068

**Published:** 2019-09-12

**Authors:** Luigi F. Cuturi, Alessia Tonelli, Giulia Cappagli, Monica Gori

**Affiliations:** ^1^Unit for Visually Impaired People, Istituto Italiano di Tecnologia, Genoa, Italy; ^2^Fondazione “Istituto Neurologico Casimiro Mondino” (IRCSS), Pavia, Italy

**Keywords:** crossmodal associations, multisensory, perception, angles, audio-visual, geometry, mathematics education

## Abstract

Developmental studies have shown that children can associate visual size with non-visual and apparently unrelated stimuli, such as pure tone frequencies. Most research to date has focused on audio-visual size associations by showing that children can associate low pure tone frequencies with large objects, and high pure tone frequencies with small objects. Researchers relate these findings to coarser association, i.e., less precise associations for which binary categories of stimuli are used such as in the case of low versus high frequencies and large versus small visual stimuli. This study investigates how finer, more precise, crossmodal audio-visual associations develop during primary school age (from 6 to 11 years old). To unveil such patterns, we took advantage of a range of auditory pure tones and tested how primary school children match sounds with visually presented shapes. We tested 66 children (6–11 years old) in an audio-visual matching task involving a range of pure tone frequencies. Visual stimuli were circles or angles of different sizes. We asked participants to indicate the shape matching the sound. All children associated large objects/angles with low pitch, and small objects/angles with high pitch sounds. Interestingly, older children made greater use of intermediate visual sizes to provide their responses. Indeed, audio-visual associations for finer differences between stimulus features such as size and pure tone frequencies, may develop later depending on the maturation of supramodal size perception processes. Considering our results, we suggest that audio-visual size correspondences can be used for educational purposes by aiding the discrimination of sizes, including angles of different aperture. Moreover, their use should be shaped according to children’s specific developmental stage.

## Introduction

Crossmodal correspondences are multisensory associations between sensory features of different nature (Spence, 2011; Parise, 2015). Evidence of such correspondences have been shown in everyday life and in controlled environments (Spence, 2011; Parise, 2015). In the context of audio-visual crossmodal correspondences, the association between auditory pure tone frequencies and spatial features such as visual size has been widely studied in adults. Indeed, although size is mainly experienced via haptic or visual sensory information, auditory information can also influence perceived size. For instance, when visual objects appear together with low or high pure tone frequency sounds, their perceived size can be biased: low pure tone frequency sounds can increase the perceived object’s size, while high pure tone frequency sounds can decrease it (Evans and Treisman, 2011; Tonelli et al., 2017). Along these lines, auditory frequency can also influence speed in the ability to discriminate relative size (Gallace and Spence, 2006). Similarly, sound features produced during impact collision, such as frequency and amplitude, can influence size estimation (Grassi, 2005). Several apparently unrelated pieces of auditory information can influence perception of products of everyday use (Spence and Zampini, 2006), as well as simple actions such as rubbing hands against each other (Jousmäki and Hari, 1998; Guest et al., 2002). This suggests an automatic nature of crossmodal correspondences (for a review: Spence and Zampini, 2006; Spence and Deroy, 2013), although other studies show that these phenomena rely on both top-down and bottom-up processes (Getz and Kubovy, 2018). It remains unclear when audio-visual size association emerges during development. Specifically, on one side, some studies on infants suggest that similarities between sound frequency and visual size can be perceived at 4 months of age (Peña et al., 2011) and at 6 months of age when auditory stimuli are speech-independent (Fernández-Prieto et al., 2015). Other studies indicate a later appearance of such audio-visual correspondences beyond 10 months of age (Haryu and Kajikawa, 2012) or even later at 30–36 months of age (Mondloch and Maurer, 2004). While development of the audio-visual size correspondence in the first years of life has been widely investigated, few have explored this phenomenon in primary school children aged between 6 and 11 years old. Some studies show that children do not gain this association until 11 years of age (Marks et al., 1987), while others show audio-visual and tactile-visual crossmodal correspondences in preschoolers when judging motion stimuli (Nava et al., 2016). This time window is of particular interest because specific multisensory calibration processes reach optimal integration in humans around 8 years of age, such as spatial navigation (Nardini et al., 2008) and size perception (Gori et al., 2008; Gori, 2015).

In general, some crossmodal correspondences such as those relative to size, are interpretable as an example of supramodal processes that convey the same stimulus property, and they do so independently of the encoding sensory modalities. Moreover, studies show that supramodal processes optimize visual perceptual learning (Zilber et al., 2014), indicating their prominent role in multisensory calibration.

To date, audio-visual correspondences relative to size perception in young children have been studied by means of binary sound frequencies (i.e., high vs. low), unveiling coarse-grained crossmodal correspondences (for a review, Spence, 2019). In the present study, we aim to understand how the association between pure tone frequency and visual size emerges during childhood, specifically by using a novel approach that accounts for a range of auditory and visual stimuli as circles or angles. This will reveal how the developmental stage might influence finer-grained crossmodal correspondence, and more specifically the more precise audio-visual association. At the same time, we investigate whether one can associate pure tones with a geometrical concept acquired late in childhood (e.g., Mitchelmore and White, 2000), i.e., angles. Although angle perception has received less investigation in the context of crossmodal correspondence literature (see Marks, 1987 for the association between high versus low pitch and angular versus rounded shapes), research has recently uncovered association with an implicit task in adults (Parise and Spence, 2012). There may be strong educational consequences if this is confirmed in children. Since multisensory processes take time to develop in childhood, we hypothesize that crossmodal correspondences may also depend on the developmental stage. Our results indicate that primary school children’s ability to use pure tone frequencies changes with age. While younger children associate visual size more with extreme pure tone frequencies (i.e., high and low), older children instead associate more intermediate pure tone frequencies with intermediate sizes and angle apertures. This difference suggests that finer audio-visual size correspondence might develop with age.

## Materials and Methods

### Participants

We recruited participants from a local primary school (Istituto Don Bosco - Genova, Italy). Given children’ availability, the number of participants was defined to be in the same range of previous successful studies investigating size perception across development (e.g., Gori et al., 2008, 2012; Petrini et al., 2014). In a matching task, we tested 66 children (35 males; all right-handed excepted 3) who ranged in age from 6 to 11 years old (6 years old, *n* = 10; 7 years old, *n* = 9; 8 years old, *n* = 10; 9 years old, *n* = 20; 10 years old, *n* = 9; 11 years old, *n* = 8). This study was carried out in accordance with the recommendations of the local health service (Comitato Etico, ASL 3, Genova, Italy) with written informed consent from all participants or their legal representatives. The study obtained written informed consent from the parents of the participants in this study in accordance with the Declaration of Helsinki. The protocol was approved by the local health service, Comitato Etico, ASL 3, Genova, Italy.

### Stimuli

Auditory stimuli were pure tones of different frequencies (250, 500, 1000, 2000, 5000 Hz) that we generated using the Audacity software^1^ at a sample rate of 44100 Hz. We used a one loudspeaker (Sony^TM^ - SRS-X11) positioned in front of the participant at a distance of ∼25 cm to present stimuli. The sound level plus background noise for all sounds was ∼70 DB. Each auditory stimulus lasted 2 s. Considering the high rate of potential task withdrawal in children, to guarantee we could compare performance between participants, we pseudo-randomized the presentation order once and used this fixed order for all participants. However, all participants completed the full set of trials. We recorded responses with a touch screen 21.5″ monitor (Dell^TM^ SX2210TB; screen resolution: 1920 × 1080; refresh rate: 60 Hz) that we positioned in front of the participant at ∼40 cm.

**FIGURE 1:**
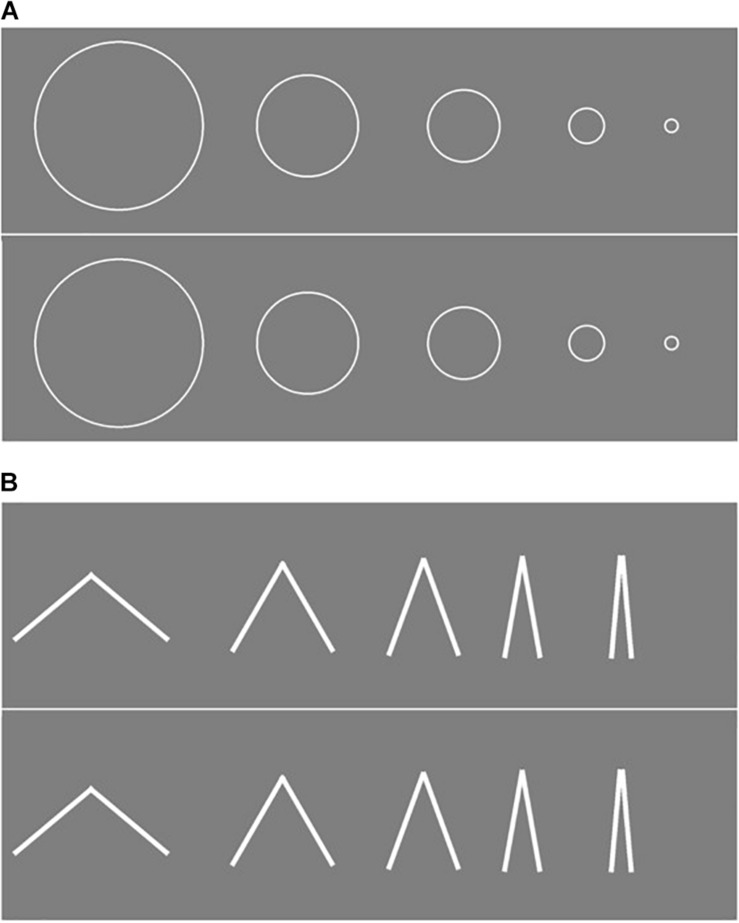
Visual stimuli used in the task. **(A)** From the left, the circle diameters correspond to 10.8, 6.5, 4.7, 2.3, and 0.8 cm. **(B)** From the left, the angles were: 100, 60, 40, 20, and 10°; each line composing the angles is 6.5 cm long. Participants were asked to indicate the visual stimulus that corresponds to the auditory stimulus they heard.

Visual stimuli consisted of circles of different sizes (10.8, 6.5, 4.7, 2.3, and 0.8 cm in diameter, see Figure 1A) or angles of different degrees (10, 20, 40, 60, and 100°, see Figure 1B).

### Design

During each trial, we presented participants with two identical arrays of five visual objects – one above the other (see Figure 1) – to test whether participants indicated upper or lower stimuli as a function of sound frequency. We introduced this aspect as a control measure, considering high pure tone frequency stimuli can be associated with elevated visual stimuli, and low pure tone frequency stimuli can be associated with bottom visual stimuli (Pratt, 1930). The participant’s response would therefore not depend on the relative position of the object, either upper or lower, in comparison to other visual stimuli on the same row. In each trial, we presented participants with a single pure tone selected from a range of different pure tone frequencies from low to high frequency (250, 500, 1000, 2000, and 5000 Hz).

The participants’ task was to select the visual stimulus that matched the auditory stimulus by touching the stimulus on the monitor. The two visual conditions (circles and angles) were randomly interleaved within a single block of trial so that each participant performed both visual conditions (circles and angles). Each stimulus level repeated eight times per condition, leading to 80 trials in total presented within one block of trials lasting a total of ∼10 min within the same block of trials.

## Statistical Analysis

To test whether children associated different sound frequencies with different visual sizes, we analyzed the influence of auditory stimulus on the chosen object separately for the circle and the angle conditions. Considering the discrete nature of the response and five possibilities to choose from, we used a multinomial logistic regression approach (via the “ordinal” package in R; Christensen, 2015) for each condition. Our protocol provided us with categorical observations, making linear models based on the decomposition of sums of squares (e.g., ANOVA) inappropriate. This led us to use an analogous and more general measure: *deviance*. *Deviance* has been defined for generalized linear models and contingency tables with categorical observation (Christensen, 2015). We performed the analysis of deviance (ANODE, i.e., analogous to the ANOVA) on the cumulative link model by taking sound stimulus (expressed as 5 levels, see Methods section) and age (expressed as 6 age groups, see Methods section) as factors. This analysis returns tables based on Wald χ^2^-tests and provides tables with type III hypothesis, which is independent of the number of observations for each factor and their combination (Kuznetsova et al., 2017). We performed *post hoc* pairwise comparisons using the “emmeans” package in R: by reporting the data on a latent-variable scale. This analysis allowed us to compute and contrast predicted probability distributions relative to each response level. We considered pairwise comparisons with *P* < 0.05 to be significant (Tukey’s corrected *p*-values are reported).

To examine whether the selection of each visual stimulus in response to each auditory stimulus depends on age, we performed a correlation analysis between age and responses wherein we treated age as a continuous variable by using decimal values. Regarding participants’ responses, for each participant, we calculated the proportion (from 0 to 1) for which they indicated each circle size/angle aperture in response to each audio stimulus level. The data then went through logit-based transformation. We performed 25 (5 sound frequencies × 5 response levels) correlations per condition (*Bonferroni* corrected *p*-values are reported).

## Results

**FIGURE 2:**
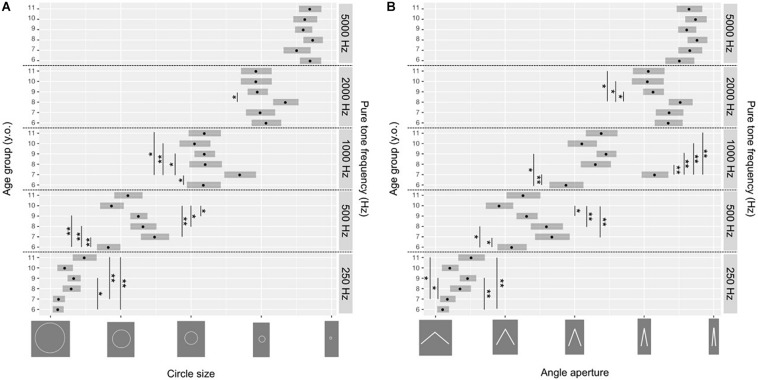
Results of all participants in the circle **(A)** and angle **(B)** condition. Each data point indicates the average of the probability distributions of each response rating corresponding to the circle (**A**; see Table 1) and angle (**B**; see Table 2) response options. Error bars indicate the 95% confidence interval. ^∗^*p* < 0.05, ^∗∗^*p* < 0.01 as reported in Table 3 (for the circle condition) and Table 4 (for the angle condition).

Table 1 presents results for the circle condition and Table 2 for the angle condition. As shown in Figures 2A,B, participants of all age groups associated high pure tone frequency with smaller circles or angles and low pure tone frequency with larger circles or angles. In the circle condition, analyzing deviance (ANODE) showed us that there was a significant effect given by the pure tone frequency (χ^2^_(4)_ = 120.11, *p* < 0.0001), the age group (χ^2^_(5)_ = 24.57, *p* < 0.001) and the interaction of the two factors (χ^2^_(20)_ = 49.9, *p* < 0.001). In the angle condition, we also observed significant effects of pure tone frequency (χ^2^_(4)_ = 122.21, *p* < 0.0001), as well as the age group (χ^2^_(5)_ = 26.91, *p* < 0.0001) and the interaction of the two factors (χ^2^_(20)_ = 56.54, *p* < 0.001). *Post hoc* analysis revealed significant differences between age groups and in both conditions, especially for the lowest and intermediate pure tone frequencies (Figure 2 presents significant comparisons; Table 3 reports for the circle condition and Table 4 for the angle condition).

**TABLE 1:** Results for the circle condition.

**Pure tone**	**Age group**	**Mean.class**	**SE**	**asymp.LCL**	**asymp.UCL**
**(Hz)**	**(years old)**				
250 Hz	6	1.11	0.04	1.03	1.19
	7	1.12	0.04	1.03	1.21
	8	1.30	0.07	1.17	1.43
	9	1.34	0.05	1.24	1.43
	10	1.21	0.06	1.10	1.32
	11	1.49	0.09	1.31	1.66
500 Hz	6	1.83	0.09	1.66	1.99
	7	2.49	0.10	2.29	2.69
	8	2.32	0.09	2.14	2.51
	9	2.26	0.06	2.13	2.38
	10	1.87	0.09	1.70	2.04
	11	2.11	0.10	1.91	2.31
1000 Hz	6	3.18	0.12	2.94	3.42
	7	3.70	0.12	3.47	3.92
	8	3.21	0.12	2.98	3.44
	9	3.19	0.08	3.05	3.34
	10	3.06	0.11	2.84	3.28
	11	3.19	0.12	2.96	3.42
2000 Hz	6	4.07	0.11	3.86	4.28
	7	3.99	0.11	3.78	4.20
	8	4.35	0.09	4.17	4.53
	9	3.95	0.07	3.80	4.09
	10	3.93	0.11	3.71	4.15
	11	3.93	0.11	3.71	4.15
5000 Hz	6	4.70	0.08	4.55	4.85
	7	4.51	0.10	4.32	4.70
	8	4.74	0.07	4.60	4.88
	9	4.60	0.06	4.48	4.73
	10	4.63	0.09	4.46	4.80
	11	4.70	0.08	4.54	4.86

*Mean.class indicates the average estimates of the probability distribution of each visual stimulus. SE indicates standard error. Lower and upper 95% confidence intervals are given by asymp.LCL and asymp.UCL, respectively.*

**TABLE 2:** Results for the angle condition.

**Pure tone**	**Age group**	**Mean.class**	**SE**	**asymp.LCL**	**asymp.UCL**
**(Hz)**	**(years old)**				
250 Hz	6	1.11	0.04	1.02	1.19
	7	1.18	0.06	1.06	1.29
	8	1.36	0.08	1.21	1.51
	9	1.47	0.06	1.35	1.58
	10	1.21	0.06	1.09	1.33
	11	1.52	0.10	1.33	1.71
500 Hz	6	2.10	0.11	1.89	2.31
	7	2.68	0.13	2.43	2.93
	8	2.60	0.12	2.37	2.83
	9	2.32	0.08	2.16	2.47
	10	1.92	0.10	1.72	2.12
	11	2.26	0.12	2.02	2.51
1000 Hz	6	2.88	0.12	2.64	3.13
	7	4.16	0.10	3.96	4.35
	8	3.30	0.11	3.09	3.52
	9	3.46	0.07	3.31	3.60
	10	3.11	0.11	2.90	3.32
	11	3.39	0.12	3.16	3.62
2000 Hz	6	4.35	0.10	4.15	4.55
	7	4.37	0.10	4.17	4.56
	8	4.53	0.09	4.35	4.7
	9	4.13	0.08	3.99	4.28
	10	4.05	0.12	3.82	4.28
	11	4.07	0.12	3.84	4.29
5000 Hz	6	4.51	0.11	4.30	4.72
	7	4.66	0.09	4.49	4.84
	8	4.77	0.07	4.62	4.91
	9	4.62	0.07	4.49	4.75
	10	4.75	0.08	4.59	4.90
	11	4.65	0.09	4.47	4.84

*Mean.class indicates the average estimates of the probability distribution of each visual stimulus. SE indicates standard error. Lower and upper 95% confidence intervals are given by asymp.LCL and asymp.UCL, respectively.*

**TABLE 3:** Age comparison for the circle condition.

**Pure tone**	**Comparison**	**Estimate**	**SE**	***z*-ratio**	***p***
**(Hz)**	**(age groups)**				
250 Hz	6–9	–1.31	0.43	–3.02	0.0308
	6–11	–1.80	0.47	–3.84	>0.01
	7–11	–1.70	0.47	–3.60	>0.01
500 Hz	6–7	–1.22	0.27	–4.46	>0.001
	6–8	–0.93	0.26	–3.62	>0.01
	6–9	–0.81	0.22	–3.71	>0.01
	7–10	1.13	0.27	4.19	>0.001
	8–10	0.84	0.25	3.31	0.0118
	9–10	0.72	0.22	3.35	0.0104
1000 Hz	6–7	–0.94	0.32	–2.90	0.0431
	7–9	0.91	0.27	3.34	0.0109
	7–10	1.16	0.32	3.62	>0.01
	7–11	0.92	0.32	2.89	0.0450
2000 Hz	8–9	0.92	0.30	3.04	0.0288

*Estimate and z-ratio <0 indicate that the visual stimulus assigned to the sound frequency was larger for the target age group (factor on the left in the comparison column) regarding comparison age (factor on the right in the comparison column). SE indicates standard error. Only significant comparisons are reported (*p* < 0.05).*

**TABLE 4:** Age comparison for the angle condition.

**Pure tone**	**Comparison**	**Estimate**	**SE**	***z*-ratio**	***p***
**frequency**	**(age groups)**				
250 Hz	6–9	–1.69	0.46	–3.72	>0.01
	6–11	–1.83	0.50	–3.71	>0.01
	7–9	–1.16	0.39	–2.97	0.0358
	7–11	–1.30	0.44	–2.98	0.0339
500 Hz	6–7	–0.85	0.26	–3.31	0.0120
	6–8	–0.73	0.24	–3.01	0.0314
	7–10	1.14	0.27	4.29	>0.001
	8–10	1.03	0.25	4.05	>0.001
	9–10	0.62	0.21	2.94	0.0385
1000 Hz	6–7	–1.84	0.30	–6.15	>0.0001
	6–9	–0.78	0.21	–3.72	>0.01
	7–8	1.27	0.26	4.89	>0.0001
	7–9	1.06	0.22	4.73	>0.0001
	7–10	1.53	0.27	5.66	>0.0001
	7–11	1.15	0.26	4.43	>0.001
2000 Hz	8–9	0.91	0.31	2.95	0.0370
	8–10	1.06	0.35	3.03	0.0297
	8–11	1.04	0.35	2.96	0.0366

*Estimate and z-ratio < 0 indicate that the visual stimulus assigned to the sound frequency was greater for the target age group (factor on the left in the comparison column) regarding comparison age (factor on the right in the comparison column). SE indicates standard error. Only significant comparisons are reported (*p* < 0.05).*

**FIGURE 3:**
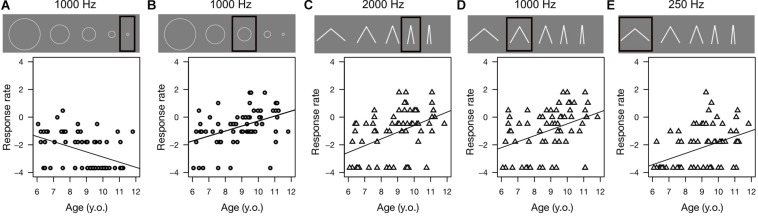
Correlation for selected frequencies and stimulus levels. Each panel shows the relationship between perceived size (represented as the proportion of indicating one of 5 visual stimuli) and participants’ ages. This figure only shows the significant correlations. Circle symbols are used for the circle condition and triangle symbols for the angle condition. For each panel, a black frame highlights the indicated circles and angles with respect to the other visual stimuli. **(A)** Represents the proportion of indicating the smallest circle in response to a 1000 Hz stimulus (*R* = −0.43, *p* < 0.01). **(B)** Represents the proportion of indicating the middle circle in response to a 1000 Hz stimulus (*R* = 0.42, *p* = 0.01). **(C)** Represents the proportion of indicating an angle of 20° in response to a 2000 Hz stimulus (*R* = 0.44, *p* < 0.01). **(D)** Represents the proportion of indicating an angle of 40° in response to a 1000 Hz stimulus (*R* = 0.39, *p* = 0.03). **(E)** Represents the proportion of indicating an angle of 60° in response to a 250 Hz stimulus (*R* = 0.41, *p* = 0.01).

Correlation analysis showed developmental trends exclusively for intermediate frequencies. As Figure 3 demonstrates, in the circle condition, we observed a significant negative correlation for the pure tone frequency of 1000 Hz (smallest circle: *R* = −0.43, *p* < 0.01), which indicates that the younger the children, the higher the proportion of responses indicating the smallest circle. We observed a significant positive correlation for responses that indicated the intermediate circle with a sound stimulus of 1000 Hz (*R* = 0.42, *p* = 0.01). Thus, older children provided this response more than their younger peers.

In the angle condition, we observed a significant correlation for the pure tone frequency of 2000 Hz indicating an angle of 20° (*R* = 0.44, *p* < 0.01), 1000 Hz indicating an angle of 40° (*R* = 0.39, *p* = 0.03) and 250 Hz indicating an angle of 60° (*R* = 0.41, *p* = 0.01). Thus, older children gave these responses more than younger ones. Other correlations were not significant, thus we did not report them.

Additionally, we tested whether participants would preferentially indicate the stimuli on the upper or lower row as a function of pure tone frequency. In the circle condition, participants mostly selected visual stimuli from the lower row rather than the upper, with no differences depending on sound frequency (χ^2^_(4)_ = 7.96, *p* = 0.09) or age (χ^2^_(5)_ = 7.55, *p* = 0.18). In the angle condition, we observed that sound frequency (χ^2^_(4)_ = 9.95, *p* = 0.04) and age (χ^2^_(5)_ = 14.2, *p* = 0.01) had significant effects, but there was no interaction between the factors (χ^2^_(20)_ = 26.84, *p* = 0.14). However, also in this condition, the tendency was to preferentially select stimuli from the lower row (see Supplementary Materials).

## Discussion

This study investigated whether primary school children associate sound frequency with visual objects’ size. We asked children between 6 and 11 years old to indicate the size of the shape that matched the sound we presented. Visual stimuli were either circles or angles. Results show that children from 6 to 11 years of age associated high pure tone frequencies with smaller visual stimuli and low pure tone frequencies with larger ones. The main novelty of our result regards the crossmodal correspondences for intermediate sound frequencies. In particular, the correlation analysis shows a developmental trend in relation to the progressive association between sound frequency and visual objects’ size. This result suggests that brain organization of the relation between size and pure tone frequency develops with age, probably depending on maturing of supramodal processes that underlie size perception.

In choosing the sounds to test, we decided to isolate their tone frequency as the variable influencing the selection of the object’s size and angle aperture. For this reason, we did not balance loudness across the different pure tone frequencies of the auditory stimuli. This choice is grounded in the pedagogical purpose of the current study. Although we are aware of the general association between loudness and perceived size (Smith and Sera, 1992) and therefore the possible limitations of this choice, we decided to focus on pure tones of different frequencies. These could be effortlessly reproduced in conditions other than experimental, such as within the classroom. According to the Fletcher and Munson curves (Fletcher and Munson, 1933), depending on the sound level in DB, perceived loudness varies depending on sound frequency. Although this phenomenon may have influenced the lowest sound frequencies we tested (e.g., 250 Hz), we presented sounds at the level in which Fletcher and Munson curves get flattened corresponding to the tested sound frequencies (i.e., 70 DB). In other words, perceived loudness for each sound frequency was roughly equal across tested sound frequencies. Nevertheless, since loudness has been shown to influence also perceived size (Smith and Sera, 1992), future works may be pursued to investigate whether intermediate loudness levels would show developmental patterns similar to what observed with sound frequency.

Studies have observed audio-visual crossmodal correspondences for perceived size in newborns, children, adults and animals (Fitch and Hauser, 2003). This indicates the importance of such perceptual properties for communication and their possible innate rather than experience-based origin (Hinton et al., 2006). In the study presented here, we observed developmental trends in the use of intermediate sizes of circles and angles depending on pure tone frequency and participants’ age. In particular, when we presented children with the 1000 Hz stimulus, older children used more intermediate sizes and angle apertures, whereas younger ones relied more on extreme stimuli. Regarding angles, we observe the same trend for sound stimuli of 2000 Hz. Along these lines, performance of the children showed that the use of the smallest circles in response to the 1000 Hz stimulus is higher for younger compared to older children, which shows the former tend to use the extreme options more than the latter. This result indicates that the ability to use and possibly discriminate objects and angles, when associated with pure tone frequency, may develop as age advances during childhood. In this context, the development of perceived size based on multisensory cues may influence the association of size and pure tone frequency.

Previous studies have shown haptic rather than visual preference for size perception until the age of 8–10 years old (Gori et al., 2008). Up to this age, the brain is yet to calibrate the other senses to provide a reliable estimate of size, as multisensory integration is not fully developed. Similarly, the absence of recalibration may influence visual judgments of size in response to sound stimuli, especially in the case of intermediate sound frequencies. Such pure tone frequencies require finer estimation, whereas one can easily discriminate the extreme levels with broader judgments. In other words, multisensory recalibration across development might provide the brain with a size estimation system that becomes more precise as children age, thereby influencing one’s ability to “crossmodally” transfer a range of pure tone frequencies (i.e., ranging from low to high) to a scale of visually presented stimuli. Understanding the perception of size for intermediate sound frequencies contributes fundamentally to the literature because developmental studies on crossmodal correspondences for size perception have mostly taken advantage of comparing the association of size with high versus low pure tone frequencies. By focusing on the association between visual spatial frequency and auditory amplitude temporal modulation, previous studies used a range of stimuli that show how multisensory experience and top-down processes guide crossmodal associations’ development (Guzman-Martinez et al., 2012; Orchard-Mills et al., 2013), as well as their use in visuospatial attention tasks (Mossbridge et al., 2011). In our study, we focused on the range of potential perceptual associations by investigating their changes across childhood development specifically in size perception. Intermediate sound frequencies allowed us to test how possible categorization of audio-visual correspondences interplay with development. The developmental trends we observed represent a novel result that offers information surrounding the brain capacity of refining multimodal association from the first months of life onward. Brain imaging studies have observed that acoustic scales are processed in the intraparietal sulcus, but also that they are category independent. Indeed, this area is involved in supramodal processing of size rather than strictly visual (von Kriegstein et al., 2007). In this context, our study hints at development-dependent maturation of brain mechanisms that begin with the extremes of the continuum (e.g., high pure tone frequency/small vs. low pure tone frequency/large) and progressively take advantage of finer steps in the continuum, increasing in precision. Moreover, the pattern of results our study observed sheds light on the relative versus absolute debate in the context of crossmodal correspondences (Spence, 2019). As previously suggested (Parise, 2015; Spence, 2019), most studies in crossmodal correspondences using only a pair of stimuli assume a monotonic relationship between sound frequencies and size judgments. In this context, by using a range of stimulation, our results hint at developmental influence on the monotonic relationship, possibly involving a shift from absolute to relative size crossmodal correspondences with age. In this context, the current study’s methodology would be useful in disclosing the interrelationship between different stimulus features in the generation of crossmodal correspondences across development, including those interesting pitch with elevation and lightness.

Interestingly, in the case of the pitch-elevation association, natural statistics provided evidence for a strong correlation between crossmodal correspondences and the statistical spatial distribution of sounds in natural conditions (Parise et al., 2014). This finding suggests that such associations rely on environmental properties that as such generate priors leading to absolute crossmodal correspondences. Contrary to our results on perceived size, Parise and coworkers observed a non-linear relationship between pitch and elevation in the perceptual correspondences which correlates with the measured natural statistics. Although differences in the nature and range of the auditory stimuli used might have a role (i.e., pure tone frequencies from 250 to 5000 Hz in our study versus band-pass noise ranging from <800 Hz to 9000 Hz in Parise et al., 2014), natural statistics relative to pitch-size associations might differ in terms of distribution and in their relationship with experience. Given our results, further research investigating natural statistic relative to pitch-size associations would need to take into account also developmental aspects.

Altogether, our findings suggest a developmental pattern of crossmodal correspondences specifically for size perception. In the context of perceived elevation, studies on infants show that crossmodal correspondences between pitch and visual elevation are present since the first days of life (Walker et al., 2018). Lewkowicz and Turkewitz (1980) showed that prior visual stimulation shapes infants’ physiological response, in terms of heart rate, to auditory stimuli of different intensities; adults instead do not show such behavior suggesting developmental changes in the spontaneous attendance of specific stimulus’ features. The question on the emergence of audio-visual size correspondences remains therefore open; fundamental insights would come from studies with infants as well as natural statistics. Although we observed developmental trends based on a combined influence of age and stimulus range, the specific stimulus’ feature of interest seems to have a strong role, suggesting developmental pattern and changes that occur with different timing for different features of interest.

The maturation of decision making processes may underlie the developmental trends our results indicate. Developmental changes in decision making have been observed in several tasks, showing that, as they grow up, children acquire the competencies needed to reach efficient reasoning and to use heuristics to accomplish their judgments (Jacobs and Klaczynski, 2009). Considering the nature of our experimental paradigm, children in the process of indicating the visual stimulus corresponding to the heard sound had to make a choice, thereby unveiling their decisional strategies. Our results allow us to extrapolate a pattern of behavioral choices that resemble the maturation of children’s abilities to spread their choices across the given options. By using the intermediate visual stimuli in response to intermediate pure tone frequencies, older children seem to use a more conservative approach at their decisional level. This strategy might be related to their maturational stage, prompting them to act less abruptly and more consciously when they are making a choice in each trial. Simultaneously, younger children might be more uncertain in choosing the shape corresponding to the heard sound in the case of intermediate pure tone frequencies. This means they may provide a more category-based response by using the extreme visual stimuli because of their clear difference in size and angle. However, prior knowledge acquired through stimulus presentation history influences behavioral performance beginning in early childhood (e.g., 7 years old) (Sciutti et al., 2014), which excludes this aspect as a candidate process underlying the developmental changes we observed in the present study.

To provide a deeper understanding of the influence of such maturational processes, future research is necessary to examine learning related aspects like educational performance or intelligence quotient to investigate the cognitive processes underlying the development of the investigated crossmodal correspondences. Additionally, in order to provide hints on the prior information children might use to accomplish the task, it would be interesting to directly ask them on which basis they chose the shapes corresponding to the pure tones (see Saluja and Stevenson, 2018 for the association between color and taste).

In this research, we studied crossmodal correspondences about shapes (circle) and angles. This was due to the fact that young children might show difficulties understanding the concept of angles (Mitchelmore and White, 2000; Prescott et al., 2002). We observed that, in addition to circles, angular apertures are associated with pure tone frequency. This result supports the hypothesis that crossmodal correspondences about size perception are category independent, not only in the context of the auditory information (von Kriegstein et al., 2007), but also relative to the visual stimulus associated with an auditory stimulus (Parise and Spence, 2012). Future research should further investigate other graphical properties that are possibly influencing crossmodal correspondences relative to angles (e.g., by varying the length of each segment composing the angle).

Altogether, these results indicate that multisensory associations between pure tone frequencies and shapes, either angles or circles, are exploitable in educational contexts starting from 6 years old. Using multisensory information to foster learning is not new. Already at the beginning of the 20th century, Montessori proposed a teaching method that included visual, auditory, haptic and kinesthetic stimulation in the classroom (Montessori and Holmes, 1912). Considering the multisensory nature of the everyday environment surrounding children, the employment of multisensory perception for educational purposes may be useful for improving classroom learning (Shams and Seitz, 2008). Along these lines, Parrott et al. (2015) investigated how ascending and descending pitch cues influence the visual perception of slopes in bar graphs and scatter plots in adults. These findings suggest audition could be used to cue mathematical concepts generally transmitted visually. Our study followed a similar approach by unveiling the influence of the developmental stage on audio-visual size correspondence to provide scientific support for the use of sounds in educational contexts, which are based on actual behavioral measurements in children. Indeed, investigating crossmodal correspondences between audition and vision is of particular interest regarding the development of pedagogical tools for concepts like size comparisons and angle discrimination. For instance, designing technological games in educational contexts would strongly benefit from the outlined development of crossmodal correspondences in children. The developmental pattern observed in our results suggests that fine-grained crossmodal associations may be incapable of immediately helping younger children; younger children may then benefit from training with intermediate frequencies. On the other hand, older children may benefit from a range of pure tone frequencies, which they can use for dealing with more complex operations rather than pure comparisons such as in the case of addition and subtractions between angles. Particularly regarding angles, studies have already observed that angle discrimination by walking in children depends on age (Cuturi et al., 2017). This suggests that the capability of discriminating angles at a perceptual level might take time to fully develop, meaning there is a need to develop new strategies to teach difficult concepts such as angles at school. We recommend that apparently unrelated auditory information, in the form of pure tone frequency, would be useful in addition to vision to teach geometrical concepts such as size comparison and angle discrimination.

This study is framed within an EU-H2020-ICT-funded project, namely the weDRAW project^2^. This project aims to develop educational solutions that take advantage of sensory modalities other than vision (Duffy et al., 2017; Price et al., 2017; Volpe and Gori, 2019). In this context, our findings indicate that auditory pure tone frequency provides an additional cue for discriminating sizes and angles, perhaps offering one an opportunity to enhance purely visual teaching methods. Following these and previous findings regarding crossmodal correspondences, the project’s consortium has developed serious games and activities that integrate body movement with auditory information exploiting pitch to discriminate angles. Crossmodal correspondences outlined in this work may be useful for teaching concepts such as angle and size comparison in primary school by means of a multisensory approach. Such a systematic investigation of the association between a range of pure tone frequencies and visual objects including angles allowed us to test whether this association is generalized across development to different categories of shape. Our results provide the basis of a useful educational guideline for primary school teachers.

## Data Availability

The datasets generated for this study are available on request to the corresponding author.

## Ethics Statement

This study was carried out in accordance with the recommendations of the local health service (Comitato Etico, ASL 3, Genova, Italy) with written informed consent from all subjects or their legal representatives. All subjects or their legal representatives gave written informed consent in accordance with the Declaration of Helsinki. The protocol was approved by the Comitato Etico, ASL 3, Genova, Italy.

## Author Contributions

LC, AT, GC, and MG conceived and designed the study, wrote and edited the manuscript. LC performed the experiments. LC and AT analyzed the data. All authors gave final approval for publication.

## Conflict of Interest Statement

The authors declare that the research was conducted in the absence of any commercial or financial relationships that could be construed as a potential conflict of interest.

## Footnotes

1 http://audacity.sourceforge.net/

2 www.wedraw.eu
