# Supplementary material for: Coarse to Fine Audio-Visual Size Correspondences Develop During Primary School Age
Source: Front Psychol. 2019 Sep 12;10:2068. doi: 10.3389/fpsyg.2019.02068 (PMC6751278; doi:10.3389/fpsyg.2019.02068)
Supplement: Supplementary file 1 [file Table_1.pdf]

Supplementary materials

Figure S1 represents subjects' preference in indicating angles on the upper or lower row of stimuli. All data points fall between 1.5 and 2, thus indicating that most of the responses were indications of angles on the lower row. Nevertheless, we do observe a significant effect given by sound frequency and age group. Post-hoc analysis on the sound frequency (reported on table S1) indicate that, although preferences were toward the lower row, distributions of the responses after pure tone frequencies of 250Hz and 500Hz were surprisingly more towards the upper row. However, these differences are relative to the comparisons between pure tone frequencies, therefore cannot be interpreted as an absolute preference for the lower row of visually presented stimuli. Similarly, significant differences relative to age group comparisons (reported on table S2) refer to data points falling between 1.5 and 2, thus indicating that subjects across all age groups indicate more stimuli belonging to the lower rather than the upper row.

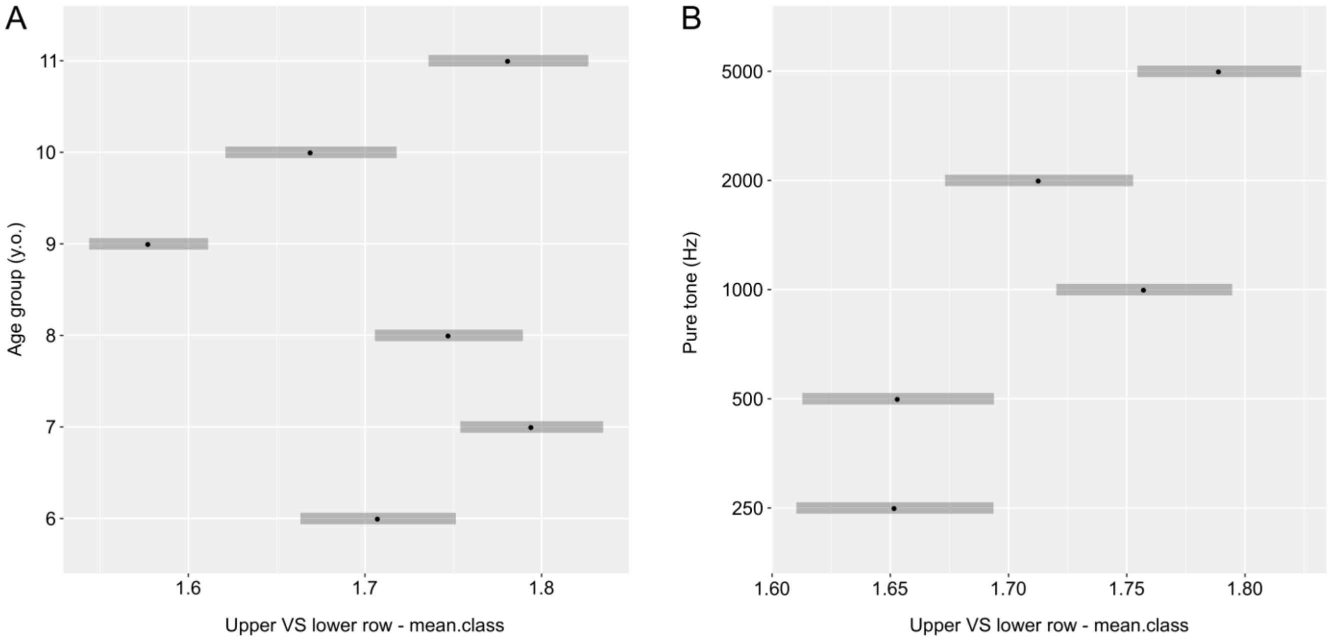

Figure S1. Preference for upper or lower row of stimuli. Each data point indicates the average of the probability distributions of each response rating corresponding to the upper (between 1 and 1.5) or lower row (between 1.5 and 2). Error bars indicate the 95% confidence interval.

Comparison (age groups)	Estimate	SE	z-ratio	p
6 - 7	-0.6	0.19	-3.146	0.0206
6 - 9	0.59	0.13	4.399	< 0.001
7 - 9	1.19	0.17	6.997	< 0.001
7 - 10	0.801	0.19	4.204	< 0.001
8 - 9	0.806	0.14	5.768	< 0.001
9 - 10	-0.389	0.13	-2.905	0.0426
9 - 11	-0.957	0.15	-6.215	< 0.001
10 - 11	-0.568	0.18	-3.223	0.016

Table S1. Estimate and z-ratio < 0 indicate that the visual stimulus assigned to the sound stimulus was on the bottom row for the target age group (factor on the left in the comparison column) regarding comparison age (factor on the right in the comparison column). SE indicates standard error. Only significant comparisons are reported ($p < 0.05$).

Comparison (pure tone)	Estimate	SE	z-ratio	p
250-1000	-0.5303	0.148	-3.581	<0.01
250-5000	-0.8176	0.165	-4.963	<0.001
500-1000	-0.496	0.151	-3.291	<0.01
500-5000	-0.7833	0.167	-4.688	<0.001
2000-5000	-0.5398	0.168	-3.222	0.0111

Table S2. Estimate and z-ratio < 0 indicate that the visual stimulus assigned to the sound stimulus was on the bottom row for the target sound frequency (factor on the left in the comparison column) regarding comparison age (factor on the right in the comparison column). SE indicates standard error. Only significant comparisons are reported ($p < 0.05$).
